# Exploration of *Solanum xanthocarpum* Schrad. & Wendl. against *Mycobacterium avium* Subspecies *paratuberculosis* and Assessment of Its Immunomodulatory and Anti-Inflammatory Potential

**DOI:** 10.3390/ph15111367

**Published:** 2022-11-08

**Authors:** Varsha Srivastava, Manthena Navabharath, Saurabh Gupta, Shoor Vir Singh, Sayeed Ahmad

**Affiliations:** 1 Bioactive Natural Product Laboratory, School of Pharmaceutical Education and Research, Jamia Hamdard, New Delhi 110062, India; 2 Department of Biotechnology, Institute of Applied Sciences & Humanities, GLA University, Mathura 281406, Uttar Pradesh, India

**Keywords:** *Solanum xanthocarpum* Schrad. & Wendl., Johne’s disease (JD), Crohn’s disease (CD), *Mycobacterium avium* subspecies *paratuberculosis* (MAP), immunomodulatory, anti-inflammatory

## Abstract

*Mycobacterium avium* subspecies *paratuberculosis* (MAP), being a dairy-borne pathogen, resistant of pasteurization and other sterilization techniques, is a major cause for development of inflammatory bowel disorders such as Johne’s disease (JD) in dairy animals and Crohn’s Disease (CD) in humans, for which no therapy is available to date. In the absence of effective therapy or a vaccine, management of CD has been accomplished by removal of the affected intestines. However, usually, even after removal of 2/3 of the intestine, CD reoccurs. Hence, there exists a need to develop an alternative therapy for such infection. The potential of herbals remains unexplored against MAP and related infections. Therefore, the conducted study is a novel initiative for the evaluation of anti-mycobacterial activity of bioactive extracts of *Solanum xanthocarpum* Schrad. & Wendl. against MAP infection. The said plant was authenticated according to the Ayurvedic Pharmacopoeia of India. Qualitative and quantitative evaluation of the extracts were done using chromatographic and spectroscopic techniques. Preliminary in vitro pharmacological assessments revealed the immunomodulatory and anti-inflammatory potential of the extracts. REMA assay was conducted to determine their anti-MAP activity along with determination of the best active extract. The hydro-alcoholic extract showed the best inhibition of MAP, providing a potential ray of hope against this emerging major pathogen of animals, and associated with Crohn’s disease and other autoimmune disorders in human beings.

## 1. Introduction

In India, *Mycobacterium avium* subspecies *paratuberculosis* (MAP) infection is endemic in domestic livestock [1]. The infection has been reported in ruminants, wild life [2,3] and human beings. Live bacilli have been recovered from milk in domestic livestock [4,5], human breast milk [6], and other milk products [5,7]. These bacilli are excreted through milk and are not inactivated during pasteurization [8], resulting in human infection. Infection has also been associated with a large number of autoimmune disorders in the human population [9]. Infection of domestic livestock with MAP also leads to huge losses in productivity [1]. Infected animals are characterized by a decline in body condition, weight loss (which might or might not be accompanied with diarrhea), loss in productivity (milk and meat) and reduced fertility. They also become susceptible to other infections due to the growth of bacilli within macrophages in the germinal centres of lymph nodes.

Diagnosis of MAP infection is difficult and involves the use of multiple tests. MAP bacilli are refractory to common antibiotics [10]. Very few studies using multi-antimycobacterial drugs in animals [11] and human beings for long periods exhibited anti-mycobacterial activity. Long term usage of modern medicines has led to drug resistance against available anti-mycobacterial drugs [12]. Not only this, in India, the cost of treatment with multiple anti-mycobacterial drugs is over the cost of animals, where native breeds of domestic livestock are known for their low productivity, making treatment unrealistic.

Even the consumption of milk and milk products made from pasteurized milk leads to MAP infection in the human population, resulting in development of IBD, including Crohn’s disease (CD), Ulcerative Colitis (UC), Irritable Bowel Syndrome (IBS) and other autoimmune disorders. Long-term utilization of multi anti-tuberculous drugs has not been practiced in developed countries [10] since prolonged use can lead to development of drug resistance against all existing anti-mycobacterial molecules [13], as happened in the case of tuberculosis (multiple drug resistance (MDR), –extensive drug resistance (XDR) and total drug resistance (TDR)). In the absence of effective therapy or a vaccine, management of CD has been accomplished in developed countries mainly by surgical intervention and removal of the affected intestines. In western countries, people prefer surgery over drug therapy. However, in many instances, even after removal of 2/3 of the intestines, CD relapses. By contrast, in India people prefer modern medicines and alternative system of medicine like ayurveda and homeopathy as the primary modes of treatment, keeping surgery as the last option. The present work is the first research to work on a model using the Ayurvedic system of medicine against MAP bacilli. Plants having anti-mycobacterial activity provide alternative courses that identify therapeutic intervention points for prevention and possible permanent remission of infection in animals (Johne’s disease) and may even help to combat those infections caused by drug-resistant pathogens.

Anti-mycobacterial drugs are less effective for the management of MAP-related disorders due to the thick lipid-rich cell wall and intracellular nature of bacilli. In view of the rapid increase in cases of animal and human infections, the demand for natural products for this chronic incurable infection is logical. We have been approached by many patients suffering from MAP infection for diagnosis and treatment over the past 20 years [14].While suggesting anti-mycobacterial drugs, we found that they are highly insufficient in the treatment of disease and tend to develop resistance. Due to the chronic nature of infection and long treatment schedule, the cost is high, which is also a major discouragement for the use of allopathic medicines in the treatment of MAP infection in animals and human beings. *Solanum xanthocarpum* Schrad. & Wendl. has been mentioned as a potent anti-inflammatory agent in Ayurveda [15]. The entire plant is said to contain a wide range of chemical substances with unique biological properties. The plant possesses diverse pharmacological properties and bioactive compounds such as phenolics, flavonoids, glucosinolates, and steroidal glycoalkaloids. [16]. It is a medicinal herb that grows untamed in the wild and is widely utilized in traditional medicine. The presence of phytochemical compounds from various plant parts such as roots, stems, leaves, fruits, and seeds has been reported to have a wide range of pharmacological activities such as hepatoprotective [17], cardioprotective, antiasthmatic, and mosquito repellent properties, according to the extensive literature. The scientific confirmation of folklore claims and its historic usage in healing various diseases is confirmed by current research on the pharmacological activity of the plant [18]. However, limited information is available about its anti-tuberculous activity and its underlying mechanism of action. Here, we investigate the anti-MAP activity of the plant, *Solanum xanthocarpum* Schrad. & Wendl., exploring its immunomodulatory as well as anti-inflammatory potential, using in vitro models that provide a ray of hope for the development of treatment choices against MAP infection using natural alternatives.

## 2. Results

### 2.1. Collection, Authentication and Extraction of Plant Materials

Plant materials were freshly collected and authenticated in-house using botanical and phytochemical reference standards following the protocol of the Ayurvedic Pharmacopoeia of India (API), examining the macroscopic as well as the microscopic characters of the plant. Samples of the authenticated plant materials having voucher numbers BNPL/JH/2020/26, BNPL/JH/2020/27, BNPL/JH/2020/28 and BNPL/JH/2020/29 for stem, leaves, fruits and roots, respectively, have been stored in the Bioactive Natural Product Laboratory, Jamia Hamdard, New Delhi depository for future reference.

#### 2.1.1. Macroscopy

*Solanum xanthocarpum* Schrad. & Wendl. is an annual herbaceous weed. Leaves are petiolate, ovate, elliptical or oblong, and hairy in appearance. Stems are prickly and hairy with visible nodes and internodes, and green in colour. The mature stems are more glabrous, with prominent furrows. Thin bark with prominent wood shows the presence of prominent pith in the centre. However, the mature and dry stem shows the presence of hollow pith with short to slightly fibrous fractures. Fruits are globular surrounded by a persistent calyx. The ripe fruits are yellow, having a few transverse wrinkles along with small rootlets. Fruits are bitter in taste. Macroscopical characters are shown in Supplemetary Figure S1.

#### 2.1.2. Microscopy

**Figure 1:**
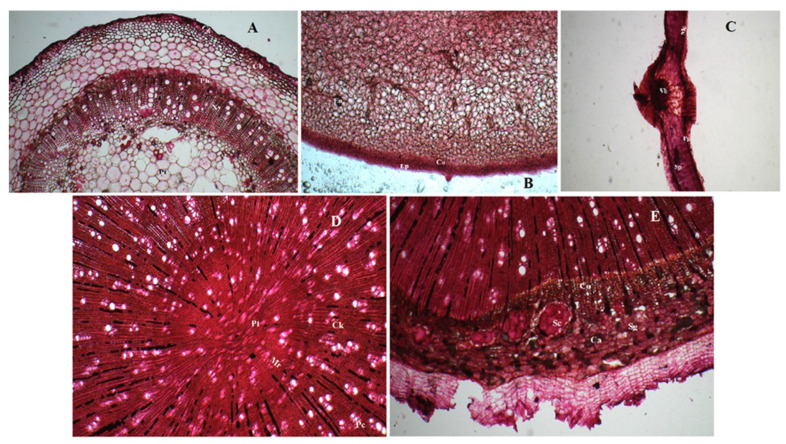
Microscopic evaluation of the whole plant of *Solanum xanthocarpum* Schrad. & Wendl. (**A**) Transverse section of stem which shows the presence cork (Ck) having thin-walled hexagonal cells followed by 6–7 layers of cork cambium (Cb). Phloem (Pb) shows the presence of fibres Xylem (Xy) shows the presence of tracheids, vessels, fibres and parenchymatous cells traversed by xylem rays. Pith (Pt) is wide, located in the centre. (**B**) Transverse section of fruit showing a single layer of epidermis (Ep) covered with a thin cuticle followed by a layer of collenchymatous cells (Co). The endosperm (En) shows the presence of oil globules. (**C**) Transverse section of leaf midrib having biconvex shape illustrating the presence of bicollateral vascular bundles (Vb), mesophyll cells (MP) along with palisade parenchyma (Pp) and spongy parenchyma (Sp). (**D**,**E**) Transverse sections of root depicting the presence of central pith (Pt), medullary rays (Mr), cork (Ck) comprised of thin-walled rectangular cells, pericycle (Pc), stone cells (Sc), calcium oxalate crystals (Ca) as sandy masses and simple starch grains (Sg) in the secondary cortex.

Transverse sections of stem showed the presence of cork with thin-walled hexagonal cells followed by 6–7 layers of cork cambium. Phloem had the presence of fibres. Xylem showed the presence of tracheids, vessels, fibres and parenchymatous cells traversed by xylem rays. Pith was found widely and located in the centre (Figure 1A).

The transverse section of fruit showed a single layer of epidermis covered with a thin cuticle followed by a layer of collenchymatous cells. The endosperm showed the presence of oil globules (Figure 1B).

The transverse section of leaf midrib has a biconvex shape illustrating the presence of bicollateral vascular bundles, mesophyll cells and palisade parenchyma and spongy parenchyma (Figure 1C).

Transverse sections of root showed the presence of central pith, medullary rays, cork comprising of thin-walled rectangular cells, pericycle, stone cells, calcium oxalate crystals as sandy masses and simple starch grains in secondary cortex (Figure 1D,E).

#### 2.1.3. Extraction of Active Constituents

Dried plant materials were powdered and extracted using aqueous, ethanolic and hydro-ethanolic (50–50 ratio) solvents using developed extraction methods. Yield of the extracts obtained were in compliance with the API (Ayurvedic Pharmacopoeia of India). The aqueous extract was found to exhibit highest percentage yield among all the three extracts (18.18 ± 0.13% *w*/*w*) followed by hydroalcoholic (14.15 ± 0.02% *w*/*w*) and alcoholic extracts (9.79 ± 0.02% *w*/*w*). All values were calculated in triplicate.

### 2.2. Phytochemical Evaluation

#### 2.2.1. Total Phenolic Content (TFC) & Total Phenolic Content (TPC)

The flavonoid contents were found to be 28.35 ± 1.9, 26.14 ± 1.61 and 16.97 ± 1.77% per milligram of hydroalcoholic, alcoholic and aqueous extracts, respectively. All the readings were calculated in triplicate.

Standard and sample readings were determined using a spectrophotometer at 765 nm. Phenolic contents were obtained as 75.49 ± 3.60, 60.66 ± 5.49, 46.69 ± 3.10% per milligram of alcoholic, hydroalcoholic and aqueous extracts respectively. All the readings were calculated in triplicate.

#### 2.2.2. Free Radical Scavenging Activity

**Figure 2:**
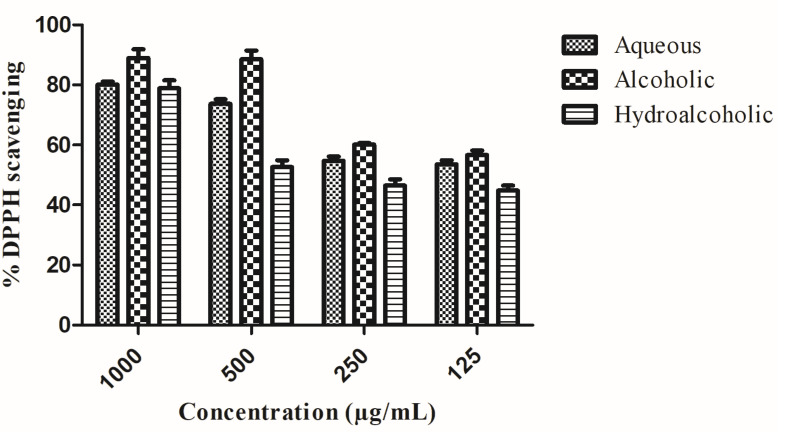
Assessment of free radical scavenging activity using the DPPH assay (% DPPH inhibition).

The alcoholic extract exhibited the greatest inhibition of reactive oxygen species (ROS) at the lowest concentrations. Percentage DPPH inhibition of all the extracts are shown in Figure 2. Half-maximal inhibitory concentration (IC_50_) value is tabulated in Table 1.

### 2.3. In Vitro Immunomodulatory and Anti-Inflammatory Activity

#### 2.3.1. Pinocytic Activity Assay

**Figure 3:**
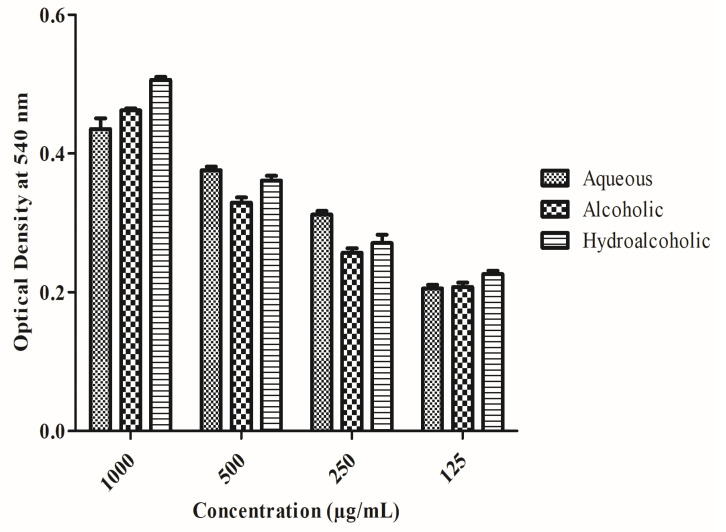
In vitro immunomodulatory assessment and action on pinocytes.

Immunomodulatory activity of the prepared extracts was assessed in vitro, and its action on pinocytes was determined using the pinocytic activity assay. Analysis of pinocytic activity was done via neutral red uptake. Results are shown in Figure 3. Hydro-alcoholic extracts showed significant action on pinocytes, followed by alcoholic and aqueous extracts, inferring its immunomodulatory potential.

#### 2.3.2. Effect of the Extract on Membrane Stabilization Assay

Anti-inflammatory activity was assessed in vitro, and extracts were found to exert protective effects. Erythrocytes, against heat induced haemolysis, showed a concentration-related effect. Haemolysis induced by heat, causing hypotonicity, was the protective effect (Table 1).

### 2.4. Confirmed Purity of Mycobacterium avium Culture and Anti-Mycobacterium Activity of Solanum xantocarpum Extracts Using the Resazurin Microtitre Assay (REMA)

**Figure 4:**
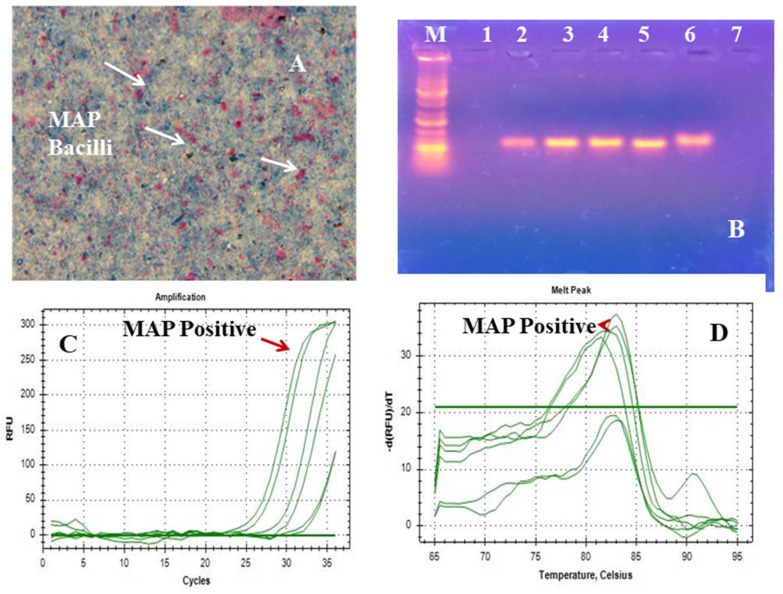
Confirmation of *M. paratuberculosis* at the molecular level. (**A**) Microscopy (Ziehl-Neelsen stain). (**B**) Conventional PCR (*IS900* 413 bp) where Lanes M–7 represents 1 kb DNA ladder, control, positive control, sample 1 (DNA from Solid culture of MAP strain ‘S5’ batch 1), sample 2 (DNA from solid culture of MAP strain ‘S5’ batch 2), sample 3 (DNA from liquid culture of MAP strain ‘S5’ batch 1), sample 4 (DNA from liquid culture of MAP strain ‘S5’ batch 2) and blank respectively. (**C**) Amplification of RT-PCR (*IS900* 413 bp); (**D**) Melt Peak of RT-PCR (*IS900* 413 bp).

The Polymerase Chain Reaction (PCR) based method using insertion sequence IS900, is considered specific for the rapid and specific detection of MAP globally. The insertion sequence IS900 is a 1451-bp segment that lacks inverted terminal repeats and does not generate direct repeats in target DNA (Figure 4). Microscopy showed pink-colored, short rods indistinguishable with *Mycobacterium avium* subspecies *paratuberculosis* on acid fast staining using the Ziehl Neelsen stain (Figure 4A). Agarose gel electrophoresis (Figure 4B). showed specific amplification of gene (IS900) using primer sequences P90B AND P91B (Figure 4C). The melting-curve of the real-time PCR *ISO900* (413) bp gene is shown in Figure 4D.

**Figure 5:**
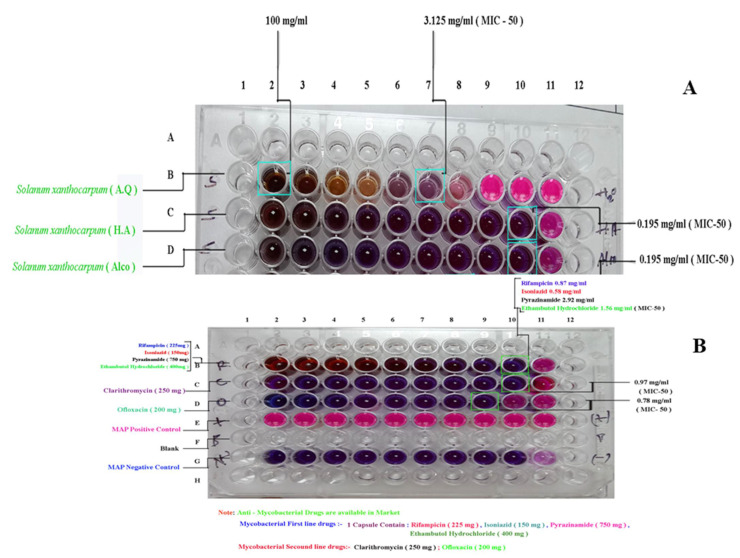
REMA assay. (**A**) In vitro activity of test extracts, i.e., A.Q (aqueous), H.A (hydro-alcoholic) and Alco (alcoholic) extracts of *Solanum xanthocarpum* Schrad. & Wendl. (**B**) In vitro activity of standard anti-mycobacterial first line drugs, i.e., Rifampicin (225 mg), Isoniazid (150 mg), Pyrazinamide (750 mg), Ethambutol hydrochloride (400 mg) and second line drugs, i.e., Clarithromycin (250 mg), Ofloxacin (200 mg).

Anti-MAP activity was assessed in vitro, and the activity of plant extracts was determined using the REMA assay. Percent inhibition of MAP is shown in Figure 5 and was measured as Minimum inhibitory concentration (MIC_50_) (Table 1). Both the alcoholic and hydroalcoholic extracts showed maximum inhibition. MIC_50_ was found to be 0.195 mg/mL for both the alcoholic as well as the hydroalcoholic extracts.

### 2.5. Quantitative Validation of Solamargine, Solasonine & Solasodine

**Figure 6:**
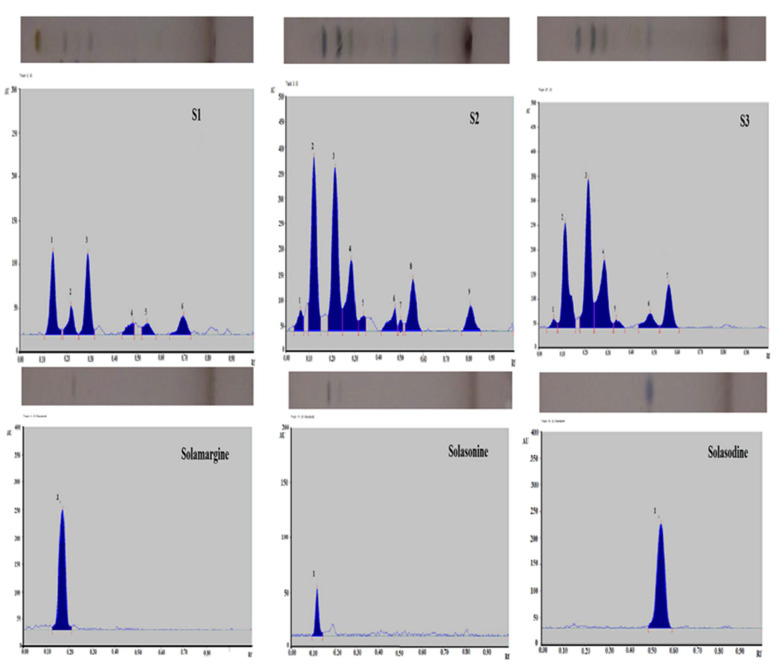
TLC photographic representation and quantitative estimation of biomarkers Solamargine, Solasonine Solasodine in *Solanum xanthocarpum* Schrad. & Wendl. aqueous (S1), ethanolic (S2) and hydro-ethanolic (S3) extracts respectively at 540 nm post derivatization using butanol: ethyl acetate: 10% acetic acid (5:3.5:1.5).

For simultaneous quantitative estimation of Solamargine, Solasonine and Solasodine, a TLC plate was developed in a twin-trough chamber containing Butanol: ethyl acetate: 10% acetic acid (5:3.5: 1.5 *v*/*v*/*v*) as the solvent system. Densitometric scanning was performed at 540 nm after derivatizing with anisaldehyde-sulphuric acid reagent (since the compounds were UV-inactive). The method developed was linear ranging from 200–2000 ng/spot with regression coefficients (r^2^) 0.9796, 0.9994, and 0.9761 for Solamargine, Solasonine and Solasodine, respectively (Table 2). The amounts of standards Solamargine, Solasonine and Solasodine were quantified as maximum at 4.48, 5.84 and 1.35% *w*/*w* respectively in hydroalcoholic; 4.65, 8.69 and 1.47% *w*/*w*, respectively, in alcoholic and 0.10, 2.26 and 0.56% *w*/*w* in aqueous extracts of *Solanum xanthocarpum* Schrad. & Wendl. (Table 2). Photographic representation and quantitative estimation of biomarkers along with chromatograms are shown in Figure 6.

## 3. Discussion

MAP, commonly referred to as paratuberculosis (Para-TB, a chronic inflammatory condition occurring predominantly in the ruminant population. It is endemic at a global level [4]. Reports suggest that about 72% of dairy cattle are infected with these bacilli. Microbiological tests including IS900 PCR, d & I Enzyme Linked Immunosorbent Assay, Latex Agglutination Test (LAT), the immunofluorescence antibody test (I_FAT) and culture showed 21.4–71.4%, 10.7–42.1% and 9.0–60.0% bio-load in milk powder, flavored milk and liquid milk retailed in local marketplace by leading Indian brands [1]. With a prevalence rate of >30.0% in humans, MAP bacilli enter the system causing a wide variety of incurable autoimmune disorders such as rheumatoid arthritis (RA), inflammatory bowel disorders (IBD), thyroiditis, Alzheimer’s disease (AD), Parkinson’s disease, and Type I & Type II diabetes mellitus in human beings [7]. The bacilli are also excreted in the environment with faeces and in milk. Because of their sub-clinical and slow-growing nature, these pathogenic bacilli are often ignored. Since the bacilli are resistant to chemical and thermal sterilization techniques such as chlorination and pasteurization [19], their transmission and control in human beings has become difficult. Anti-tuberculous drugs are given, but are insufficient, leaving surgical removal of intestines as the last alternative. However, relapse occurs in the cases of surgery as well. To date, no therapy is available in allopathic as well as the Ayurvedic system of medicine. *Solanum xanthocarpum* Schrad. & Wendl. has been reported to encompass diverse pharmacological properties. It is described as anti-inflammatory (*Jvara*) in traditional scripture [16]. It also contains various bioactive compounds such as steroidal glycoalkaloids, phenolics, flavonoids, and glucosinolates. Since the disorders caused by MAP are of an inflammatory nature, this potent anti-inflammatory weed was chosen for the study. The present study screened for the best active extract having anti-MAP activity. All the three extracts (aqueous, ethanolic and hydro-ethanolic) were qualitatively analyzed for the metabolic characterization of major bioactive phytoconstituents i.e., phenols, flavonoids and free radical scavenging compounds. Flavonoids and phenols are reported to be active against *Mycobacterium tuberculosis* [20,21], and MAP, belongs to the same genus; hence, determining the concentration of flavonoids and phenols became essential for meeting the rationale of the study. Some studies suggest that inflammation occurs due to free radical production; hence, the presence of free radical scavenging compounds may provide an additional avenue towards the control of this pathogen [22]. The greatest amount of flavonoids was obtained in the hydroalcoholic extract of the plant i.e., 28.35 ± 1.9%. Phenols (75.49 ± 3.60%) and free radical scavenging compounds (IC_50_ value: 15.16) were higher in the alcoholic extract. Extracts were found to be rich in flavonoids, phenols and free-radical scavenging compounds. Along with the qualitative analysis, the extracts were estimated quantitatively for the presence of metabolites using chromatography. Steroidal glycoalkaloids (Solasodine, Solasonine and Solamargine) are the major phytoconstituents of *S. xanthocarpum* having anti-inflammatory and immunomodulatory activities [23]. Hence, the major plant biomarkers (S1, S2 and S3) were quantified along with development of well-defined HPTLC fingerprints. Estimation of Solamargine, Solasonine and Solasodine was done using butanol: ethyl acetate:10% acetic acid (5:3.5: 1.5 *v/v/v*) as the mobile phase. The highest amounts of major biomarkers were quantified in the alcoholic and hydroalcoholic extracts of the plant. The qualitative and quantitative estimation of the extracts helped in determining the quality of the procured extracts. Pinocytic activity is a measure of immunity. The higher the pinocytic activity, the higher the immunomodulation [24]. The membrane stabilization assay signifies anti-inflammatory activity [25]. The greater the membrane stabilization, the greater the anti-inflammatory activity. Hence, preliminarily pinocytic activity and the membrane stabilization assay were also assessed. The pinocytic assay signified the immunomodulatory potential of the prepared extracts. Hydro-alcoholic extracts showed significant action on pinocytes, followed by the alcoholic and aqueous extracts, inferring its immunomodulatory potential. The membrane stabilization assay depicting anti-erythrogenic potential showed higher anti-inflammatory potential of hydroalcoholic and alcoholic extracts. Since, no therapy is available against MAP to date, the potential of the plant was assessed in vitro using the REMA assay [26]. In vitro anti-MAP activity using the REMA assay showed that the minimum concentration of the extract required to inhibit the growth of MAP was 0.195 mg/mL, exhibited by alcoholic and the hydroalcoholic extracts.

Based on the observations, the hydro-alcoholic extract of the plant (SXHA) was inferred as the best active extract against MAP. It was concluded that there was no significant difference in the anti-MAP activity between the hydro-alcoholic and alcoholic extracts in spite of there being higher concentration of phenols and flavonoids in the alcoholic extracts. Therefore, it was assumed that there were other metabolites responsible for the predicted anti-MAP activity. Hence, the hydroalcoholic extract was selected as the BAE, (even though flavonoids and phenols were lower compared to other extracts) having a comparable anti-MAP activity. In the AYUSH system of medicine, hydroalcoholic extracts are more acceptable and safer in comparison to alcoholic extracts. Hence, considering the experimentations and the observations, the BAE was chosen.

## 4. Methodology

### 4.1. Materials and Chemicals

The standard biomarkers (Solasodine, Solasonine and Solamargine) were obtained from Sigma-Aldrich, St. Louis, MO, USA. All the solvents used for analysis were HPLC-grade (Merck Specialities Private Limited, Mumbai, India. Middlebrook 7H9 medium procured from Sigma-Aldrich, St. Louis, MO, USA. The Indian Bison Type strain of mycobacteria was procured from the Biotechnology department, GLA University, Mathura, India.

### 4.2. Plants Collection and Authentication

Whole plant parts were freshly collected from the farm area of the local Veterinary University, Mathura (Uttar Pradesh, India). The collected plant materials were authenticated using botanical and phytochemical reference standards as per the protocol mentioned in API, and samples were deposited in Bioactive Natural Product Laboratory at Jamia Hamdard, New Delhi, India.

The leaves, fruits, stems and roots of the plant were macroscopically and microscopically examined. A transverse section was prepared and stained, and microscopy was conducted using the method proposed by Kokate (2010).

### 4.3. Extraction of Bioactive Compounds

The dried plant parts were powdered and extracted using aqueous, ethanolic and hydro-ethanolic (50:50 ratio) solvents using in-house designed extraction methods to obtain three extracts of the plant [27].

### 4.4. Phytochemical Estimation

#### 4.4.1. Estimation of TFC and TPC

Ten milligrams of each of the plant extracts were reconstituted in 1 mL of the respective solvents in which they were extracted and vortexed for 45 min followed by centrifugation for 10 min at 13,000 RPM at room temperature. Clear supernatants were collected and stored for further analysis.

TFC of the respective plant extracts were determined using aluminum chloride [28]. Total flavonoid content was calculated as mean ± SD (*n* = 3). A calibration curve was plotted using a standard (rutin) and the results were expressed as mg rutin equivalent per gram of the dried plant extract.

TPC of the plant extracts were determined using the Folin and Ciocalteu method as per the reported method [29] with slight modifications. Sample and standard readings against blanks were taken at 765 nm. Phenolic contents were calculated as gallic acid equivalents (GAE/g) of the dried plant material based on the plotted standard curve of gallic acid (10–1000 µg/mL). All the determinations were done in triplicate.

#### 4.4.2. Assessment of Free Radical Scavenging Potential (DPPH Assay)

Evaluation of the plant extracts for free radical scavenging activity was done using DPPH (1,1-diphenyl-2-picryl-hydrazyl) reagent as per the protocol described in [30]. Each sample determination was done in triplicate and average IC_50_ values were calculated.

### 4.5. In Vitro Immunomodulatory and Anti-Inflammatory Activity

#### 4.5.1. Pinocytic Activity Assay

Assessment of preliminary immunomodulatory activity was done using an in vitro pinocytic assay. This is used for the determination of pinocytes [31]. In the immune system, pinocytic activity is a major function of macrophages. Peritoneal macrophages (PEM) were mixed with aqueous, ethanolic, and hydro-ethanolic extracts of *S. xanthocarpum* (125–1000 µg/mL) in wells of a 96-well plate (5 × 106 cells/well). There were also control wells that received media (no extract). After 48 h of incubation at 37 °C, the culture medium was withdrawn, and each well was filled with 0.1 percent neutral red solution (100 µL/well). Plates were incubated for another 4 h at 37 °C. The medium was withdrawn after 4 h and the macrophages were washed twice with phosphate buffer solution (PBS). A 100 µL cell lysing solution was used to further lyse the cells (10 mM Tris buffer, pH 8.0). Plates were then incubated at the same temperature for the next 2 h. Using an ELISA plate reader, the absorbance was measured at 540 nm. Absolute OD values were used to measure pinocytic activity (reflecting dye uptake) [31]. The greater the pinocytic activity, the greater the phagocytic activity and vice versa [32]. Pinocytic activity was expressed in terms of OD.

#### 4.5.2. Membrane Stabilization Assay

##### Erythrocyte Suspension Preparation

Freshly collected 10 mL human blood was transferred to 15.0 mL tubes and centrifuged for 5 min at 3000 RPM. An equal volume of normal saline solution was used to wash the blood samples thrice. Blood volume was measured and reconstituted as a suspension of concentration 10% *v*/*v* using 10mM sodium phosphate isotonic buffer solution (pH 7.4) [33].

##### Heat Induced Haemolysis (HIH)

This was performed as per the method described in [34] with slight modifications described by Gunathilake et al. [35]. Haemolysis level was calculated by the equation of Okoli et al. [34];

Percent of haemolysis inhibition = (1 − B_1_/B_2_) × 100

where:

B_1_ = absorbance of reaction control
B_2_ = absorbance of test sample

### 4.6. In Vitro Anti-MAP Activity

#### 4.6.1. Preparation of *Mycobacterium avium* Subspecies *paratuberculosis* Suspension

One microlitre (1 μL) loopful of MAP culture from Middlebrook 7H9 medium procured from Sigma-Aldrich, St. Louis, MO, USA (without antibiotic) was taken using a sterilized loop. Fresh culture aged up to 2 weeks giving positive confirmation of MAP (stored at room temperature or 37 °C) was used. Fresh growth was added to 5 mL of Middlebrook 7H9 medium in a sterilized screw cap bottle containing 6–8 glass beads. The suspension was vortexed for 15–20 s for homogenization. Large clumps were allowed to settle by standing for 10–15 min. The obtained supernatant was used as the test suspension. Concentration of the supernatant was adjusted to a 0.5 McFarland standard (1% Barium Sulfate 0.5 mL in 1% sulphuric acid 99.5 mL) by visualization. Middlebrook 7H9 medium was used to dilute the supernatant (if concentrated). More growth was added if the supernatant’s turbidity was low. A 0.5 McFarland standard suspension corresponds to approximately 10^7^ cfu/mL for MAP bacilli [36], 10^8^ cfu/mL for other mycobacteria [37] and for other bacteria [38]. The suspension was diluted accordingly. Dilutions were made in a series of 1:10 (0.1 mL of suspension into 0.9 mL Middlebrook 7H9 medium) until the required concentration of 10^2^ cfu/mL was achieved.

#### 4.6.2. Confirmation of *M. paratuberculosis* at Molecular Level: Characterization by IS900 PCR

MAP cultures were characterized using procured primers both specific and unique to MAP, IS900 (P 90/91).

MAP colonies were characterized by IS*900* PCR using P90 and P91 primers as described in protocol [39]. Primers sequences used:

- Forward primer-P90B 5′-GAA GGG TGT TCG GGG CCG TCG CTT AGG-3′
- Reverse primer-P91B 5′-GGC GTT GAG GTC GAT CGC CCA CGT GAC-3′

#### 4.6.3. Resazurin Microtiter Assay (REMA)

The assay was performed as per the reported procedure in [26]. A volume of 100 µL of MAP suspension was used as inoculum per well. Plant extract solutions were prepared in 10-fold serial dilutions. Volume of 100 µL of the prepared dilutions was added to each well of sterilized Nunc^TM^ Microwell^TM^ Delta-treated 96-Well Nunclon, flat bottom microplate (Thermofisher Scientific, Waltham, MA USA). Antibiotic free growth control and MAP-free sterilized control of the medium were added to each plate. Further, 200 µL of sterilized water was added to each plate in all the outer wells during incubation to impede evaporation. Plates were covered with sterilized 96-well plate lids and incubated at 37 °C. After 14 days, each well was stained using freshly prepared 0.04% resazurin sodium salt solution (32 µL) (Resazurin SRL, Milano, Italy). Plates were re-incubated for 2 days at 37 °C. Colour transformation from blue (oxidized state) to pink (reduced state) indicates bacterial growth. MIC-50 values were calculated using an ELISA reader (i-mark Bio-Rad, Hercules, Ca, USA) to detect the lowest drug concentration that prevented colour change, which was in the range of 0.01–0.2 mg/mL.

### 4.7. Quality Control

#### 4.7.1. Thin Layer Chromatography

Precoated TLC plates were activated by running the plate in methanol followed by drying it in an oven for 5 min to remove the impurities present on the plate. Samples for application as spots on the TLC plates were prepared as 30 mg/mL (30 mg of plant extract dissolved in the suitable solvents in which the respective extracts were prepared) in water and ethanol respectively. After trying different solvent systems by trial and error, the presence of targeted compounds was confirmed by TLC in a specific solvent system. Thereby, a mobile phase using Butanol, ethyl acetate and 10% acetic acid was prepared in the ratio 5:3.5:1.5 for development of TLC fingerprints.

The prepared mobile phase was placed in clean twin-trough TLC chamber layered with a filter paper on one of the walls for better saturation, covered properly, and left undisturbed for 45 min. Prepared samples were spotted on the pre-activated TLC plates and allowed to develop in the pre-saturated chromatographic chamber. The plate was allowed to develop until the solvent front reached up to 80%. The plate was then dried. Standard bio-markers were not visible in white light, 254 nm and at 366 nm (UV inactive). The developed plates were dipped in freshly prepared anisaldehyde sulphuric acid reagent (10 mL Glacial acetic acid + 5 mL H_2_SO_4_, added to 85 mL methanol followed by 0.5 mL Anisaldehyde and mixed well), and dried in an oven for 5 min until bands were visible.

#### 4.7.2. High Performance Thin Layer Chromatography (HPTLC)

HPTLC of the prepared extracts was performed and simultaneous analysis of different biomarkers was carried out using an HPTLC method developed in-house [27]. HPTLC fingerprints of the plant extracts were established by developing suitable solvent systems using thin layer chromatography for their separation. The solvent system showing best resolved spots were selected for HPTLC.

One milligram (1 mg) each of Solamargine, Solasonine and Solasodine were dissolved in HPLC grade methanol to a concentration of 1000 µg/mL. A sample of 30 mg/mL of each extract was also prepared. A precoated silica gel 60 F254 aluminium sheet was used for sample application. Quantitative estimation of all the three extracts was performed using authenticated standards from Sigma Aldrich. Butanol:ethyl acetate:10% acetic acid (5:3.5:1.5) was used as the solvent system for simultaneous quantification of Solamargine, Solasonine and Solasodine.

A volume of 2.0 µL of each sample was kept in the width of the track at 4.0 mm band length on pre coated silica gel G 60 F254 plates (E. Merck, 0.20 mm thickness) using Linomat V (HPTLC sample applicator). After sample application, plates were developed up to 80 mm through a saturated glass chamber with a suitable solvent system. Precoated aluminium sheets (20 × 10 cm for *S. xanthocarpum* extracts) with silica gel 60F254 of thickness 0.2 mm were used on which spots of three extracts were applied in duplicate using a Linomat 5 applicator attached to the HPTLC system. A calibration plot was developed using concentrations of the standards (Solamargine, Solasonine, and Solasodine) in the range of 0.2–2 ng/mL. The developed plates were scanned and quantified post-derivatization using anisaldehyde-sulphuric acid reagent at 540 nm with WINCATS software installed on the system.

## 5. Conclusions

The study followed an exhaustive approach in screening and selection of the BAE with anti-MAP activity. Since no work has been done to test the potential of herbal plants against incurable *Mycobacterium avium* subspecies *paratuberculosis* infection of animals, and possible therapeutic management in infected individuals, the study is novel and helped in determining the anti-MAP potential of a local medicinal herb of AYUSH system of medicine (*Solanum xanthocarpum*). A hydroalcoholic extract of the plant showed promising results. However, a lot more work needs to be done to explore the bioactive extracts at the molecular level, identifying the responsible metabolites for such activity.

## Figures and Tables

**Table 1:** In vitro pharmacological analysis of aqueous, alcoholic and hydroalcoholic extracts of *Solanum xanthocarpum* Schrad. & Wendl.

	*Solanum xanthocarpum* Schrad. & Wendl.			
S. No.	Biological Activity	Aqueous Extract (SXAQ)	Hydro-Alcoholic Extract (SXHA)	Alcoholic Extract (SXA)
1	IC_50_ value against free radicals (DPPH inhibition)	25.22	28.71	15.16
2	MIC_50_ value against MAP activity mg/mL	3.125	0.195	0.195
3	IC_50_ value against heat-induced haemolysis	239	139	209

The most active plant extract was finalised and reported as SXHA. Anti-MAP (MIC_50_) was found to be 0.195 mg/mL for both SXHA and SXA extracts.

**Table 2:** Quantification and validation of Solamargine, Solasonine, Solasodine in different extracts of *Solanum xanthocarpum* Schrad. & Wendl.

Validation Parameters			
Parameters	Solamargine	Solasonine	Solasodine
Wavelength	540	540	540
Linearity range (ng/spot)	20–2000	20–2000	20–2000
Regression equation	*y* = 1.893*x* + 2033.5	*y* = 1.3154*x* + 36.701	*y* = 1.4892*x* + 1086
Regression coefficient	0.9796	0.9994	0.9761
Slope	1.893	1.3154	1.4892
LOD (ng/spot)	14.59	11.51	5.76
LOQ (ng/spot)	24.24	34.89	17.45
Precision (% RSD)	0.265	0.665	0.235
Drug content recovered	112.08–112.31%	101.29–102.83%	95.6–111.8%

## Data Availability

Data is contained within the article and supplementary material.

## Supplementary Materials

The following supporting information can be downloaded at: https://www.mdpi.com/article/10.3390/ph15111367/s1. Supplementary Figure S1: Macroscopic evaluation of whole plant of *Solanum xanthocarpum* Schrad. & Wendl; (A) stem, (B) whole plant (leaf, stem and fruit), (C) fruit. (D, E) root.

## Abbreviations

g: gram; mL, millilitre; UV, ultra-violet; µL, micro litre; µg, microgram; mg, milligram; OD, optical density; DPPH, 2,2-Di–phenyl-1-picryl hydrazyl; PEM, REMA, resazurin micro-titre assay; TLC, thin layer chromatography; HPTLC, high performance thin layer chromatography; AU, area unit.
