# Supplementary material for: Exploration of Solanum xanthocarpum Schrad. & Wendl. against Mycobacterium avium Subspecies paratuberculosis and Assessment of Its Immunomodulatory and Anti-Inflammatory Potential
Source: Pharmaceuticals (Basel). 2022 Nov 8;15(11):1367. doi: 10.3390/ph15111367 (PMC9693291; doi:10.3390/ph15111367)
Supplement: Supplementary file 1 [file pharmaceuticals-15-01367-s001.zip › pharmaceuticals-1642005-Supplementary.pdf]

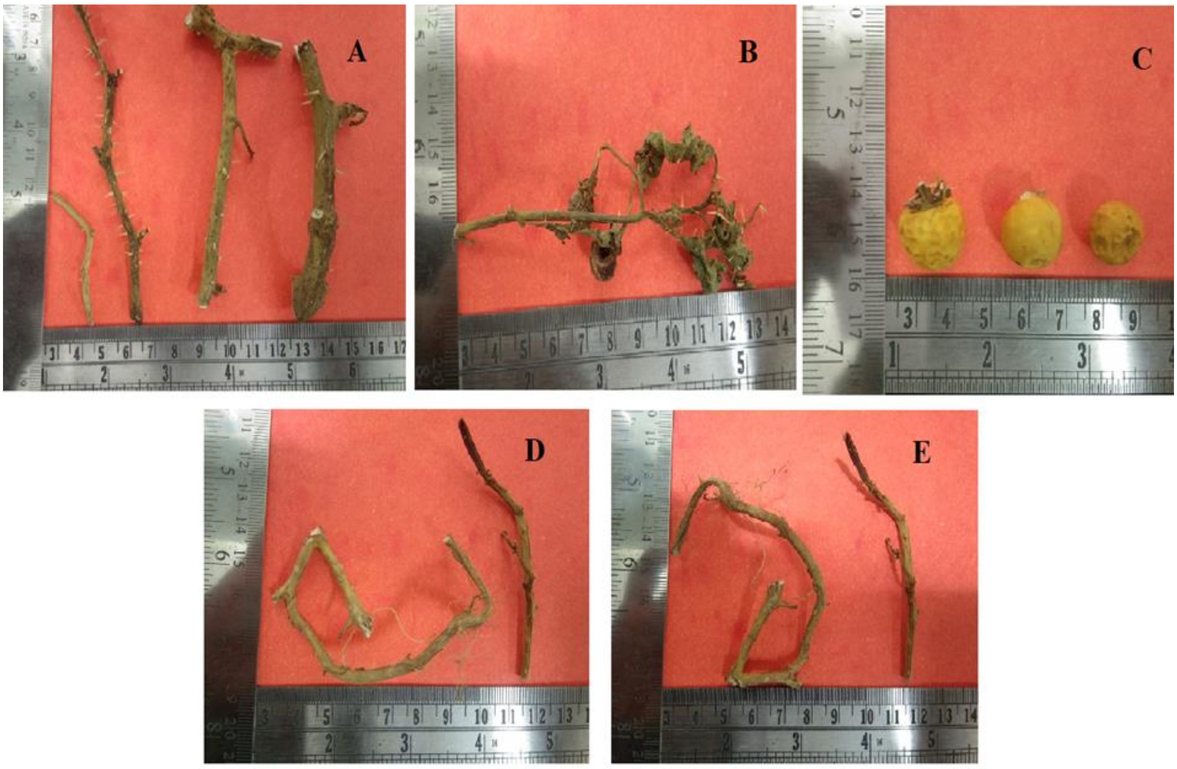

Figure S1. Macroscopic evaluation of whole plant of *Solanum xanthocarpum* Schrad. &Wendl; (A) stem, (B) whole plant (leaf, stem and fruit), (C) fruit. (D, E) root.
